# Turning over New Leaves

**Published:** 1889-08-31

**Authors:** 


### Turning Over New Leaves.

(Books for Review should be sent to The Editor, The Lodge, Porchester Square, W.)

### DAPHNE'S DARING.*

"Daphne's Daring," by Mrs. Phillips, is a conventional love story, which deserves commendation for being neither unduly sensational nor emotional. The story is far from original, but it is told with a pleasant directness and simplicity. Oliphant Grey is a manly and attractive hero, consistently well drawn, but the other characters are mere sketches, insufficiently worked up and with little individuality. Exception to this must be made in the case of Miss Lottie Barton, an old maid of the best type. The whole book is very slight, and it would be unfair to criticise it too gravely, but we must express our opinion that Mrs. Lee is an improbable sort of woman in the class to which she is supposed to belong, and that the elopement which gives the title to the book was a highly unnecessary proceeding.

Mrs. Phillips is severe upon English girl schools. Of Effie Maitland she writes: "She was wise in boarding-school conceit. She had graduated at a first-class establishment in Brighton, where girls in their walks learnt the 'use of the eyes' as well as the use of the globes, and ended in being 'finished' specimens of some English girlhood, in whom there is any amount of guile."

If the main incidents in the story are somewhat worked out, it nevertheless possesses no little merit, and is a very wholesome book.

* "Daphne's Daring: a Love Story." By Mrs. A. Phillips. Joseph Hughes, Pilgrim-street, Ludgate-hill, E.C. 1s.

